# Job Strain and Mental Health Among Older Adults: Results From the National Health Interview Survey

**DOI:** 10.1093/geroni/igaa057.547

**Published:** 2020-12-16

**Authors:** Christina Hatten, Melissa DuPont-Reyes, Tailisha Gonzalez, Rosenda Murillo, Sandra Echeverria, Elizabeth Vasquez

**Affiliations:** 1 University at Albany, State University of New York, Albany, New York, United States; 2 Texas A&M University, College Station, Texas, United States; 3 CUNY School of Public Health, New York, New York, United States; 4 University of Houston, Houston, Texas, United States; 5 University of North Carolina, Greensboro, Greensboro, North Carolina, United States; 6 University at Albany (SUNY), Rensselaer, New York, United States

## Abstract

Increased life expectancy and retirement age have contributed to a gradual aging of the US workforce. Extant literature has identified job strain (e.g. job demands, work-related stressors etc.) as a risk factor for both physical and mental health outcomes in working populations. However, there is comparatively less evidence examining job strain and mental health among older working adults. We utilized National Health Interview Survey data (2015), which contained detailed information on work conditions, to investigate the relationship between fear of job loss and self-rated mental health among working adults at 25-54 and 55+ years of age. We fit multiple logistic regression models that accounted for the complex survey design. Mental health was assessed using the K6 screening instrument that measures nonspecific psychological distress. In the sample of 16,291 working adults, 11.6% worried about job loss and 13.3% reported moderate/severe mental distress. Overall, we found that the odds of poor self-rated mental health among those who reported fear of job loss were 2.35 times that of respondents with perceived job security after adjusting for age, sex, educational attainment, race and/or ethnicity, body mass index, and smoking behaviors (95% Confidence Interval (CI): 2.01-2.75). Age-stratified results showed that perceived job insecurity corresponds with a 2.33 odds of mental distress among workers aged 25-54, and a 2.44 odds of mental distress among older workers. Our findings highlight the importance of age dynamics in mental health for working adults and suggest that job strain presents a persistent challenge for the wellbeing of workers.

